# Infant Attachment and Social Modification of Stress Neurobiology

**DOI:** 10.3389/fnsys.2021.718198

**Published:** 2021-08-16

**Authors:** Katherine Packard, Maya Opendak, Caroline Davis Soper, Haniyyah Sardar, Regina M. Sullivan

**Affiliations:** ^1^Emotional Brain Institute, Nathan Kline Institute for Psychiatric Research, Orangeburg, NY, United States; ^2^Child Study Center, Child & Adolescent Psychiatry, New York University Langone Medical Center, New York University School of Medicine, New York, NY, United States

**Keywords:** attachment, amygdala, infant, corticosterone, social

## Abstract

Decades of research have informed our understanding of how stress impacts the brain to perturb behavior. However, stress during development has received specific attention as this occurs during a sensitive period for scaffolding lifelong socio-emotional behavior. In this review, we focus the developmental neurobiology of stress-related pathology during infancy and focus on one of the many important variables that can switch outcomes from adaptive to maladaptive outcome: caregiver presence during infants’ exposure to chronic stress. While this review relies heavily on rodent neuroscience research, we frequently connect this work with the human behavioral and brain literature to facilitate translation. Bowlby’s Attachment Theory is used as a guiding framework in order to understand how early care quality impacts caregiver regulation of the infant to produce lasting outcomes on mental health.

## Introduction

Our understanding of the impact of stress on neurobiology has its roots in seminal research conducted during the 1940s and 1950s. At this time, studies emerged that highlighted the impact of stress on physical and mental health (Sorokin, 1942; Scott, 1949; Wolff, 1949; Selye, 1950, 1956; Lehmann, 1952; Whitehorn, 1956; Ziskind, 1958; Bovard, 1959; Rosen, 1959). Work published soon after demonstrated that stress in early life had an even greater impact, with stress within the context of the family featured as particularly damaging – effects which became more obvious with maturation (Bowlby, 1953; Clements, 1956; Ziskind, 1958; Provence and Lipton, 1962). These effects were quickly modeled in animals across the lifespan, with remarkable convergence across species and demonstrating a robust link establishing the brain as a mediator of stress impacts (Beach and Jaynes, 1954; Fisher, 1955; Harlow and Zimmermann, 1958; Levine and Lewis, 1959; Spitz, 1960; Hess, 1962; Stanley, 1962; Denenberg, 1963, 1964; Denenberg and Whimby, 1963; Bolles and Woods, 1964; Schaffer and Emerson, 1964; Bowlby, 1965).

Over the decades, our view of stress has become more sophisticated. We now know that family support during small bouts of acute stress can provide resilience. Furthermore, individuals and communities can adapt to a vast array of harsh environments, supporting diverse human cultures in diverse climates; these adaptations can include “hidden talents” specialized for survival in such harsh environments (Ellis et al., 2020; Humphreys and Salo, 2020). On the other hand, increasing levels of chronic stress can produce increasing rates of maladaptive behaviors at levels sufficient to compromise day-to-day life, as well as compromised mental health and/or psychiatric disorders (Bos et al., 2009; Nemeroff, 2016; Perry et al., 2019a; Smith and Pollak, 2021). In this review, we focus on the developmental neurobiology of stress-related pathology during infancy and focus on one of the many important variables that can switch outcomes from adaptive to maladaptive outcome: repeated experiences with caregiver presence during infants’ exposure to chronic stress vs. stress without this social context. While this review relies heavily on rodent neuroscience research, we frequently connect this work with the human behavioral and brain literature to facilitate translation.

We place this review within a historical context to build upon the rich framework that has guided much of developmental research. While Freud can be credited with transforming childhood into a scientific framework, John Bowlby’s Attachment Theory shifted the emphasis from maternal care satisfying the infant’s “sex drive” for healthy development (Freud, 1929) to an infant social biological drive to remain with the caregiver (Bowlby, 1965, 1969). Importantly, this paradigm shift was made possible by intensive integration in the historical writing of Suttie (1999) on the importance of mother-infant social engagement, clinical observations (Bowlby, 1953, 1978, 1984; Spitz, 1960) and the animal research by American psychologists and European ethologists (Harlow and Zimmermann, 1958; Lorenz, 1958; Hess, 1962; Hinde and Spencer-Booth, 1970). Altogether, this elevated the study of child development to a scientific discipline operationally defined and with testable hypotheses. Here, we limit ourselves to a few critical features of Attachment Theory in order to link animal research and the specialized role of social context (i.e., the attachment figure/caregiver) during exposure to acute or prolonged stress in early life.

The paradigm shift of Attachment Theory highlights the value of taking a comparative approach to studying development across species and considering evolutionarily conserved features of attachment. Across species, infants work to maintain contact with the attachment figure, with evolution and survival placing heavy selection bias on infants seeking proximity to the caregiver. This observation was explained by Bowlby as the infant possessing a “drive” to remain with the caregiver. Harlow originally characterized the mother as a source of “contact comfort” for the infant non-human primate, although later observations noted that the mother did not need to provide comfort to be approached. Specifically, observations in the Harlow lab showed that infants attach to an abusive caregiver, including cases of severe and frequent attacks when the infant repeatedly tries to maintain contact with the source of maltreatment (Seay et al., 1964; Arling and Harlow, 1967). Of course, similar cases are too often documented in children (Bowlby, 1984; Crittenden, 1992; Carlson et al., 2014; Bryant, 2016) but also widely seen in chicks, dogs, and rodents (Hess, 1962; Stanley, 1962; Salzen, 1970; Rajecki et al., 1978) as well as in non-human primates (Harlow and Harlow, 1965; Maestripieri et al., 1999; Sanchez et al., 2001; Suomi, 2003; O’Connor and Cameron, 2006). However, while attachments to caregivers are learned regardless of quality of care, the quality of care does determine the quality of the attachment, as discussed below in more detail. As we search for a better understanding of this unique developmental system, animal research has attempted to go beyond black box explanations for the infant’s approach to the caregiver by taking a mechanistic approach to define neural mechanisms. We direct the readers to comprehensive reviews on the neurobiology for typical and abuse related attachment learning across species (Rajecki et al., 1978; Insel and Young, 2001; Feldman, 2017; Granqvist et al., 2017; Opendak et al., 2017; Perry et al., 2017).

## Children Use Their Parent as a Source of Safety, in Part Through Social Buffering

While presenting Attachment Theory, Bowlby noted that infants and children often showed some fear when placed in a new situation (i.e., vacation) or when experiencing a slight threat (i.e., a frightening toy), but the fear was greatly attenuated if the parent was present (Bowlby, 1978). This phenomenon, which began to be termed “social buffering,” was concurrently being shown in other species, including rats (Davitz and Mason, 1955; Stanton and Levine, 1990; Suchecki et al., 1993; Sullivan and Perry, 2015), guinea pigs (Hennessy et al., 2006), non-human primates (Coe et al., 1978; Levine et al., 1978; Mendoza et al., 1978; Sanchez et al., 2015), and humans (Gunnar and Donzella, 2002; Tottenham, 2015). Infant social buffering is robust and wanes with maturation, although social buffering continues into adolescence and adulthood in both humans and rats (Eisenberger et al., 2011; Hornstein and Eisenberger, 2017; van Rooij et al., 2017; Robinson-Drummer et al., 2019). Furthermore, as will be discussed below, there are two critical features concerning the development of social buffering. First, when the quality of care received can impact the pup’s ability to use the mother as a stress buffer, it can impact early life programming. Second, the neural circuitry supporting social buffering changes across development. For extended reviews of social buffering, please see manuscripts cited above, as well as these reviews (Gunnar and Donzella, 2002; Gunnar et al., 2007; Hennessy et al., 2009; Hostinar et al., 2014; Gunnar et al., 2015; Sanchez et al., 2015; Kiyokawa and Hennessy, 2018).

## Neural Network Supporting Social Buffering

Rodent research has identified the mechanism by which the mother can attenuate or block the infant stress response. Building off previous work on the infant and adult stress, or hypothalamic-pituitary-adrenal (HPA) axis, rodent literature showed that maternal presence (or even the maternal odor alone) blocks stress hormone release at the level of hypothalamic paraventricular nucleus (PVN), a brain area critical for initiating and coordinate the action of the HPA response, with input from diverse brain areas (Flak et al., 2014). In our lab, we assessed the network for social buffering using maternal presence while pups received 0.5 mA shocks to the foot or tail (postnatal day – PN12–14). While it was first shown that maternal presence blocked pups’ corticosterone (CORT) release (Stanton et al., 1987; Suchecki et al., 1993), we replicated and extended this work to document some of the neurobiology (Moriceau and Sullivan, 2006; Shionoya et al., 2007). This demonstrated maternal suppression of PVN activity, with microdialysis showing that maternal presence blocked norepinephrine (NE) release into the PVN. The importance of this circuit was further probed by exogenously overriding maternal presence by either infusing NE into the PVN or infusing CORT into the amygdala, which prevented social buffering. Conversely, we were able to mimic the effects of maternal presence in pups experiencing threat alone by pharmacologically blocking CORT in the amygdala or blocking NE release into the PVN (Shionoya et al., 2007). Interestingly, rat pups also decrease their mother’s CORT levels, illustrating the bidirectional regulation of physiological functions within the caregiver-infant dyad (Walker et al., 2004).

## Social Transmission of Safety Under Threat: Suppression of CORT

**FIGURE 1:**
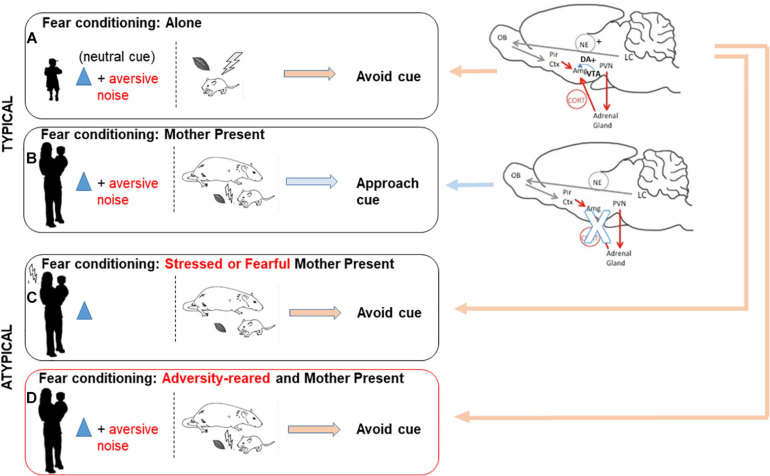
Typical and disrupted maternal social buffering of amygdala-dependent fear. During a sensitive period across species, maternal presence is an important social cue to infants: maternal suppression of infant stress hormone levels helps guide pups’ response to stimuli as safe or threatening to influence what is learned about the world. Here we illustrate two opposing brain networks and behavioral outcomes engaged by infants in response to a threat, depending on maternal presence and context. **(A)** When young are exposed to a neutral cue paired with an aversive cue, amygdala engagement promotes future avoidance of the neutral cue. **(B)** Maternal presence during a sensitive period suppresses the circuitry engaging the amygdala, including the HPA axis and dopamine from the VTA. **(C)** When the mother is stressed or fearful toward a neutral cue, buffering is impaired and amygdala-dependent learning is maintained. **(D)** Finally, maternal presence fails to buffer amygdala-dependent learning if the young has been reared in the context of adversity with a stressed or maltreating caregiver.

The maternal attenuation of pup CORT level impacts myriad brain areas and pups’ immediate interaction with the world. Perhaps one of the more dramatic illustrations of the power of social buffering is the mother rat’s ability to toggle fear and approach learning in pups between the ages of PN10 (age at which amygdala-dependent fear learning emerges) and PN15. Within this age range, odor-shock (0.5 mA) fear conditioning produces a subsequent attraction to the conditioned stimulus (CS) odor if the mother is present but produces avoidance of the CS if pups were conditioned alone. This has been causally linked to maternal suppression of CORT and amygdala suppression *via* CORT reduction and suppressed dopamine release (Sullivan et al., 2000a; Moriceau et al., 2006; Thompson et al., 2008; Barr et al., 2009; Opendak et al., 2019). This has recently been replicated in children: using a fear conditioning paradigm, children exhibited a preference for the CS if conditioned with the mother, but exhibited CS aversion if conditioned alone (Tottenham et al., 2019). These results provide a clear demonstration across species that the mother can control her offsprings’ threat response (see Figure 1).

## Social Transmission of Fear: Increase in CORT

Additional studies across species have demonstrated that the mother can mold the infant’s response to express and learn fear to a threat as needed. Clinically, the cross-generational expression of fear is well-documented: parents with PTSD can transmit specific fear to their children (De Rosnay et al., 2006; Chang and Debiec, 2016; Debiec and Olsson, 2017). Capitalizing on the power to define mechanisms in rodents, Debiec and Sullivan (2014) characterized how if a mother rat expresses fear to a learned odor CS in the presence of her pups, her pups will learn to fear that odor. The mechanism for this social transmission involved the mother releasing a fear pheromone upon exposure to the fear-eliciting CS, which in turn initiates a cascade involving activation of the pup’s Gruenberg ganglia olfactory sensory organ, increased systemic CORT and engagement of the amygdala to support learning fear to the CS odor (see Figure 1). Interestingly, exposure to the fearsome odor does not result in freezing behavior by the mother, rather, the mother demonstrates active strategies to protect pups (e.g., burying pups, covering up odor port) (Rickenbacher et al., 2017). Although there is divergence across species in the specific neural pathways engaged by the stressed mother to produce fear in infants, these studies demonstrate that mothers are nevertheless able to produce similar stress responses in their offspring.

## Quality of Care Impacts Attachment and Infant Ability to Use Mother as Social Buffer

While children attach to their caregiver regardless of the quality of care received, poor quality care is associated with compromised attachment and lasting psychosocial deficits, including increased vulnerability to psychiatric disorders. The mechanisms linking this poor care to later outcomes appear to involve impaired regulation of the infant stress system. For example, early life maltreatment and other forms of extreme poor parental care produces enhanced stress hormones in childhood, which sometimes switches to reduced stress hormone levels in later life (Gunnar and Donzella, 2002; Gunnar and Quevedo, 2007; Perry et al., 2017, Perry et al., 2019b). There is also emerging evidence that children are not socially buffered by parents in some types of unhealthy attachment dyads between parent and child (Nachmias et al., 1996).

In order to study these impacts on a more mechanistic level, we integrated our animal model of regulation with dysfunctional regulation in humans. We used a model of Scarcity-Adversity Rearing with low resources [mother has insufficient bedding for nest building (for review, see Perry et al., 2017; Walker et al., 2017)]. In this model, the mother still nurtures pups but frequently handles pups roughly while repeatedly building a nest. Importantly, pups still show robust attachment to the mother rats that treated them roughly, replicating abusive attachment observed in many species (Harlow and Harlow, 1965; Suomi et al., 2008; Raineki et al., 2010). However, this type of rearing appears to degrade the value of maternal signals to pups: maternal odor produces attenuated approach and attenuated neural responses throughout the brain (Raineki et al., 2010). Furthermore, following Adversity-Rearing, maternal presence fails to block pup fear learning (Moriceau et al., 2009). Circuit analysis showed that activation of the ventral tegmental area (VTA) is not buffered by maternal cues and these cues fail to block amygdala plasticity (Opendak et al., 2018, Opendak et al., 2019).

Recent work identifies specific features of maternal presence and behavior that compromise maternal buffering of the pup threat circuitry. We observed that during adversity-rearing, the mother fails to regulate pup cortical oscillations in response to nurturing behaviors such as grooming and milk ejection (Opendak et al., 2020). These effects were stress-hormone dependent, as blocking pup stress hormones during adversity-rearing restored maternal regulation of oscillations, as well as pup attachment behaviors. The role of stress hormones was also demonstrated in a parallel series of studies which isolated the effects of stress, maternal presence, and adverse maternal behavior (Raineki et al., 2019). Whereas the mother typically regulates acute stress responses in the infant, repeated stress in the presence of the mother produced attachment deficits and amygdala dysfunction. Notably, repeated stress alone (no mother) was not sufficient to mimic the effects of adversity rearing. This is not the result of fear conditioning, as maternal presence as been robustly shown to block fear learning *via* blockade of amygdala plasticity, preventing the pup from learning aversion to the mother (Sullivan et al., 2000b; Moriceau et al., 2006; Opendak et al., 2018; Opendak and Sullivan, 2019).

Cross-species work has also demonstrated how caregiver regulation during threat is linked to attachment quality. In the canonical Strange Situation Procedure, behavioral cues on behalf of the child, such as the child’s response to a reunion with a caregiver and how effectively the stressed infant can be soothed by the caregiver can reflect the quality of attachment (Ainsworth and Bell, 1970; Dozier et al., 2008). Adapting this test for rodents permitted both behavioral parallels between species, such as disorganized reunion behaviors, but also revealed that reunion with the mother failed to regulate cortical oscillations in adversity-reared pups (Opendak et al., 2020). Beyond this paradigm, the child’s brain shows oscillatory responses to maternal cues and the robustness of these responses is correlated with the quality of attachment (Pratt et al., 2019). This has also been seen in fMRI as a decreased response to maternal cues in the amygdala (Callaghan et al., 2019; Tottenham, 2020). Overall, these studies help identify promising biomarkers of later-life psychopathology following adversity, as well as generate testable hypotheses in children.

## What Is the Value of Social Buffering Between the Infant and Mother?

Acute stress exposure in early life has a critical role in supporting daily neurobehavioral function and permits the mother to guide pup behavior in their immediate environment, especially with respect to safety and threat. Furthermore, elevated stress hormones are well documented to disrupt brain development and social buffering can protect the developing brain from this exposure. This may be particularly important in environments where life outside the nest can be stressful, while back in the parents’ care, this stress can be reduced and a feeling of safety restored.

## What Are the Implications of Reduced Social Buffering Following Maltreatment?

Impaired social buffering results in elevated stress hormone levels in the parents’ presence – a situation unlikely to occur within typical rearing under baseline conditions. While most of the literature on chronic stress elevation does not distinguish between social and non-social context, nor directly compare the outcome of each, overall, chronic elevation of stress hormones during early life is well-documented to disrupt brain development and adult behavior. The effects of stress can extend beyond behavioral cues to include changes such as abnormal functioning, volume, and even degradation in structures such as the amygdala and hippocampus (McEwen, 2008; Hanson and Nacewicz, 2021). In children specifically, early adverse experiences can lead to lasting issues with emotion regulation (Shonkoff and Garner, 2012). This present review weaves in another level at which pups’ stress response is important: disrupted attachment with the caregiver is disrupting the pups’ ability to use the caregiver as a safety signal and depriving the infant of social guidance about safety and threat through stress hormone manipulation. The fact that poor care spares attachment but impacts the pups’ ability to use the attachment figures for stress regulation suggests maltreated pups are not only impacted by the maltreatment itself but have a double hit – maltreatment also devalues the attachment figure as a safe haven or safety signal.

**FIGURE 2:**
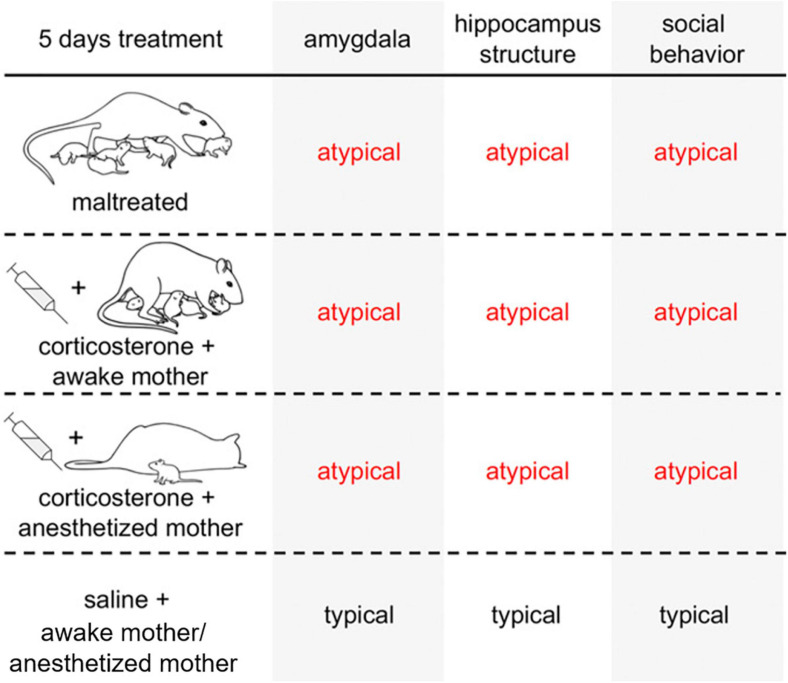
Stress with the mother present impacts amygdala and social behavior, while stress alone impacts the hippocampus. Summary of behavioral and neural effects of maltreatment and corticosterone injection paired with a social context (awake or anesthetized mother). Maltreatment impacts both the hippocampus and amygdala. The effects of maltreatment on the hippocampus can be mimicked simply by repeatedly injecting pups with corticosterone, regardless of whether the adversity occurred in a social context. On the other hand, the effects of maltreatment on the amygdala required a social context that was independent of maternal behavior: stress hormone increased within the context of a maltreating mother, a nurturing mother, or an anesthetized mother all produced similar outcomes on the amygdala and social behavior.

We conducted a series of studies in order to define the impact of chronic stress hormone elevation within a social versus non-social context (see Figure 2). We compared pups in the following conditions: reared with the Scarcity-Adversity model (induced harsh treatment by the mother), reared with a typical nurturing mother and injected with corticosterone to mimic the stress hormone increase induced by maltreatment, or reared with a nurturing mother and injected with saline (control group). After 5 days of treatment (PN8–12), maltreated and control-reared + corticosterone-injected pups showed deficits in social behavior, amygdala function, and amygdala and hippocampus volume compared to control-reared, saline-injected pups. Chronically treating pups of the same age with corticosterone while they were in the presence of an anesthetized mother, mimicked the social behavior deficits and abnormalities in the hippocampus (volume) and amygdala (c-Fos expression after a social behavior test, volume, neurogenesis, oscillations). In contrast, corticosterone treatment when pups were alone only targeted the hippocampus and did not produce social deficits (Raineki et al., 2019). Overall, these studies showed that social context of stress was necessary for producing deficits in the amygdala and social behavior and the hippocampal changes induced by elevated stress hormones did not appear to be sensitive to the social context. Interestingly, maternal behavior was not critical in these outcomes of social context on the amygdala and social behavior, since they could be produced even when the mother was anesthetized. These results suggest that compromised social buffering in early life (i.e., chronic elevated stress in a social context) may specifically target the amygdala to perturb social behavior.

In conclusion, the wide phylogenetic representation of attachment across altricial species supports the use of cross-species analysis to understand human attachment and its disruption. While the neural impact of maltreatment appears to impact much of the brain, here we highlight one facet of impacts on the infant: it increases infant baseline stress hormone levels and diminishes the attachment figure’s ability to modulate the infant’s stress response to the environment. Along with changes occurring during maltreatment, these specific outcomes begin to deconstruct the complex experience and impact of maltreatment to define one pathway of disrupted immediate behavior, as well as enduring brain programming when social buffering becomes compromised.

## Author Contributions

KP, MO, CDS, HS, and RMS wrote and revised the manuscript. All authors contributed to the article and approved the submitted version.

## Conflict of Interest

The authors declare that the research was conducted in the absence of any commercial or financial relationships that could be construed as a potential conflict of interest.

## Editor’s Note

Arun Asok edited the article in collaboration with Eric R. Kandel, Columbia University, United States.

## Publisher’s Note

All claims expressed in this article are solely those of the authors and do not necessarily represent those of their affiliated organizations, or those of the publisher, the editors and the reviewers. Any product that may be evaluated in this article, or claim that may be made by its manufacturer, is not guaranteed or endorsed by the publisher.
