# Erratum: Infant Attachment and Social Modification of Stress Neurobiology

**DOI:** 10.3389/fnsys.2021.773624

**Published:** 2021-10-05

**Authors:** Frontiers Production Office

**Affiliations:** Frontiers Media SA, Lausanne, Switzerland

**Keywords:** attachment, amygdala, infant, corticosterone, social

Due to a publisher error, the Editor's note was not included in the original article; this has now been corrected.

The original article has been updated.

